# Uveal Melanoma: A European Network to Face the Many Challenges of a Rare Cancer

**DOI:** 10.3390/cancers11060817

**Published:** 2019-06-13

**Authors:** Sophie Piperno-Neumann, Jose Maria Piulats, Matthias Goebeler, Iain Galloway, Iwona Lugowska, Jürgen C. Becker, Pia Vihinen, Joachim Van Calster, Theodora Hadjistilianou, Rui Proença, Jose Maria Caminal, Muriel Rogasik, Jean-Yves Blay, Ellen Kapiteijn

**Affiliations:** 1 Department of Medical Oncology, Institut Curie, 75005 Paris, France; 2 Department of Medical Oncology, Catalan Cancer Institute, IDIBELL, CIBERONC, Hospitalet de Llobregat, 08908 Barcelona, Spain; jmpiulats@iconcologia.net; 3 Department of Dermatology, University Hospital Würzburg, 97080 Würzburg, Germany; Goebeler_M1@ukw.de; 4 European Patient Advocacy Group: Melanoma Patient Network Europe & OcuMel, Birmingham B13 8ET, UK; iain@idcapture.co.uk; 5 Department of Melanoma, Maria Skłodowska Curie Institute-Oncology Centre, 02-034 Warsaw, Poland; Iwona.Lugowska@coi.pl; 6 Translational Skin Cancer Research, University of Essen-Duisburg, German Cancer Consortium (DKTK), 47057 Duisburg, Germany; j.becker@dkfz.de; 7 Department of Oncology and FICAN West Cancer Center, Turku University Hospital, 20521 Turku, Finland; Pia.Vihinen@tyks.fi; 8 Department of Ophthalmology, UZ Leuven, 3000 Leuven, Belgium; joachim.vancalster@uzleuven.be; 9 Department of Ophthalmology, Università degli Studi di Siena UNISI, 53100 Siena, Italy; hadjistilian@unisi.it; 10 Onco-Ophthalmology National Reference Centre, CHUC, 3075 Coimbra, Portugal; rdproenca@gmail.com; 11 Department of Ophthalmololgy, Bellvitge University Hospital, L’Hospitalet de Llobregat, 08907 Barcelona, Spain; jmcaminal@bellvitgehospital.cat; 12 EURACAn coordination team, Centre Léon Bérard, 69008 Lyon, France; muriel.rogasik@lyon.unicancer.fr; 13 EURACAN network coordinator, Department of Medical Oncology, Centre Léon Bérard, Université Claude Bernard Lyon 1, 69100 Villeurbanne, France; jean-yves.blay@lyon.unicancer.fr; 14 Department of Medical Oncology, LUMC, 2333 ZA Leiden, the Netherlands; H.W.Kapiteijn@lumc.nl

**Keywords:** uveal melanoma, rare cancer, European Reference Network

## Abstract

Uveal melanoma (UM) is the most frequent primary ocular cancer in adults, accounting for 5% of all melanomas. Despite effective treatments for the primary tumour, up to 50% of UM patients will develop metastasis, leading to a very poor prognosis and a median overall survival of 6 to 12 months, with no major improvements in the last 30 years. There is no standard oncological treatment available for metastatic UM patients, and BRAF/MEK and immune checkpoint inhibitors show disappointing results when compared to cutaneous melanoma (CM). Recent advances in biology, however, identified specific gene and chromosome alterations, potentially permitting an actively tailored surveillance strategy, and dedicated clinical studies. Being a rare cancer, UM patients have to overcome issues such as identifying referral centres, having access to information, and partnering with oncologists for specific management strategies and research priorities. Here, we describe how the European Rare Adult solid Cancer Network (EURACAN) will help in addressing these challenges and accelerating international collaborations to enhance the development of innovative treatments in UM.

## 1. Uveal Melanoma: Primary Tumour Key Points

Although uveal melanoma (UM) is the most frequent primary ocular tumour in adults, it is a rare cancer with a stable incidence in Europe of approximately 6 per million since the 1970s, and a gradient from Northern Europe, with 8 to 9 per million in Scandinavia, to Southern Europe, with less than 2 per million [1]. UM arises from melanocytes along the uveal tract: iris, ciliary body, but mainly the choroid for 85% of cases [2]. Risk factors include fair skin colour, red or blond hair, light eye colour, ocular melanocytosis and dysplastic nevi, and familial syndromes, i.e., germline BRCA1-associated protein-1 (BAP1) mutation [3,4]). Patients with germline BAP1 mutation are predisposed to familial cancers including UM, mesothelioma, cutaneous melanoma, and renal cell carcinoma, among others. Clinicians should advise genetic testing and assessment for BAP1 germline mutation in suspected patients and families [5].

In contrast to cutaneous melanoma (CM), UM does not display any UV mutation signature, and BRAF or NRAS are almost never mutated in primary UM. Moreover, UM is characterized by a very low mutational burden [6], and by activating and mutually exclusive mutations in genes encoding the G-protein-alpha subunits GNAQ or GNA11 in 90% of primary tumours [7,8]). Mutations in the CYSLTR2 or PLCB4 genes may be found in the remaining 10% [9,10]. These mutations lead to the activation of the MAPK and PI3K/AKT pathways [11] but also the transcriptional co-activator YAP through both a Hippo-independent and a Hippo-dependent circuit [12,13].

The major challenge in UM is the metastatic risk; the rate of local recurrences is below 5% but correlates with the onset of metastasis [14]. Up to 50% of UM patients will develop metastases. In 90% of cases, the liver is the first site of metastasis. The mechanism underlying hepatic tropism is not yet understood.

The main clinical prognostic factors for metastasis are tumour size, diameter and thickness, extraocular spread and ciliary body extension, and metastatic status, which are all combined in the 7th edition of the American Joint Committee on Cancer staging system [15]. The genetic features of the primary tumour provide a second risk stratification system for UM. Recurrent cytogenetic abnormalities (e.g., monosomy of chromosome 3 and amplification of chromosome 8q) have been shown as independent variables for a high risk of metastasis and a poor overall survival [16,17,18,19,20]. Gene expression profiling applying a prospectively validated, commercially available prognostic tool based on a 15-gene expression panel may help to discriminate between tumours of class 1 (low risk of metastasis) versus class 2 (high risk), but does not allow for the determination of when such evolution will occur [21]. These tools may help to tailor a surveillance strategy based on the individual risk as defined by the multidisciplinary tumour board in an expert centre, but also may allow patient selection for adjuvant studies. Surveillance of high-risk patients should focus on specific liver imaging [22]. Among imaging modalities such as liver ultrasound, computed tomography (CT), positron-emission tomography (PET)-CT, or magnetic resonance imaging (MRI), MRI shows the best sensitivity in detecting small liver lesions [23,24]. However, in the absence of randomized studies no consensus has been established yet.

So far, there is no systemic adjuvant treatment available to reduce the risk of metastasis or to improve survival in high-risk patients. Current trials in the adjuvant setting are investigating drugs such as tyrosine-kinase inhibitors (TKIs) or Met inhibitors, histone deacetylase (HDAC) inhibitors, or immunotherapy (NCT02068586, NCT02223819, NCT02068586, NCT01585194).

## 2. Metastatic Uveal Melanoma

Currently, there is no treatment available that increases overall survival in the metastatic setting [25], and there is no explanation regarding the primary resistance of UM to any systemic treatment. In the absence of standard of care treatment options, metastatic patients should be directed to clinical trials. A recent meta-analysis of metastatic UM patients enrolled in 29 phase II trials between 1988 and 2015 found very limited outcomes across all treatment groups, with a median progression-free survival (PFS) of 3.3 months and a median overall survival (OS) of 10.2 months [26].

Although extensive prospective data is missing, patients showing a limited extent of metastases seem to benefit from liver-directed therapies (e.g., hepatic intra-arterial chemotherapy [27], hepatic embolisation, radiofrequency ablation, or stereotactic radiation therapy). More recently, isolated hepatic perfusion with melphalan using a percutaneous modality has been tested in melanoma patients with promising results [28]. This selective intrahepatic delivery of high-dose chemotherapy allowed intrahepatic disease control in a retrospective study of 51 metastatic UM patients with liver-dominant disease [29] and is currently evaluated in the FOCUS randomised trial versus investigator’s choice (NCT02678572). Finally, liver surgery is associated with a survival advantage in highly selected patients achieving a microscopically complete resection of metastases [30].

To date, there are no therapies available that directly target the Gαq pathway. Recent UM trials are single drug or combination studies targeting downstream components of the MAPK pathway. The protein kinase C inhibitor (PKC) AEB071 (sotrastaurin) has been evaluated in a phase I study in 153 patients: 4 (3%) had a partial response (PR) and 76 (50%) had stable disease (SD), for a median PFS of 3.5 months [31]. In order to target effectors of the MAPK and PI3K/AKT pathways, AEB071 has been tested in combination with the MEK inhibitor binimetinib (NCT 01801358), and with the PI3K inhibitor BYL719 (NCT02273219). A second-generation PKC inhibitor is currently being evaluated in a phase I trial alone and combined with the MDM2 inhibitor HDM201 (NCT02601378). Preliminary data showed 6 PR among 66 response-evaluable patients treated with single-agent LXS196 in dose escalation, with frequent gastro-intestinal toxicities and a manageable dose-limiting toxicity hypotension in the dose-finding part of the study [32]. The MEK inhibitor selumetinib demonstrated promising results in a randomised phase II study versus chemotherapy in 101 metastatic UM patients [33]: the objective response rate (ORR) was 14% versus 0%, and the primary endpoint of PFS was improved (15.9 versus 7 weeks, *p* < 0.001). Unfortunately, the subsequent phase III SUMIT trial did not confirm the phase II results [34]. Selumetinib is currently being evaluated in an intermittent dosing schedule (NCT02768766) and in combination with paclitaxel in the SelPAc trial (EUDRACT: 2014-004437-22). The MEK inhibition with trametinib was evaluated in a single-agent phase I trial including metastatic UM patients [35] and in a randomized phase II study in combination with the Akt inhibitor GSK141795, with results indicating that it failed to provide any significant survival benefit [36]. Other TKIs (sunitinib, sorafenib, cabozantinib) targeting c-Kit or c-Met, the receptor for hepatocyte growth factor, have been investigated with modest results [37].

Therapies targeting BRAF or KIT are not indicated in UM, in the absence of the corresponding mutations. As UM patients have been excluded from large prospective trials in melanoma, small published series with anti-CTLA-4 and anti-PD-1 therapies showed low activities, with response rates of 5%, while there was no benefit with regard to progression-free or overall survival [38]. However, a small fraction of patients, potentially those displaying a high tumour mutation burden, may respond to immunotherapy [39,40].

Based on preclinical results, combination studies are running, with ipilimumab/nivolumab (NCT02626962, NCT01585194), or ipilimumab/nivolumab and radioembolization (NCT02913417).

Recently, novel immune-based approaches have tried to target more specifically the uveal tumour cells. After encouraging preliminary results [41], IMCgp100 (tebentafusp), a bispecific agent targeting the melanocyte-associated antigen gp100 by redirecting CD3+ lymphocytes, is being evaluated in a pivotal randomised phase II study versus investigator’s choice in HLA-A2-positive first-line metastatic UM patients (NCT03070392). This approach is particularly promising as UM is not only characterized by a low mutational burden, but also potential immune escape mechanisms: the eye is an immune-privileged site that could help tumour cells to escape immune elimination [42]; the tumour-infiltrating lymphocytes (TILs) cultures expanded from UM show predominant CD4+ T cells, whereas TILs from CM are CD8+ and more reactive against autologous tumours [43]. However, a subset of UM TILs may lead to anti-tumour reactivity, as tested in a first phase II study in 21 metastatic UM patients treated with lympho-depleting chemotherapy followed by a single infusion of autologous TILs and high-dose interleukine-2: 7 (35%) had an objective response of limited duration [44].

Similarly, glembatumumab vedotin, a monoclonal antibody-drug conjugate against the transmembrane glycoprotein NMB that is expressed on the surface of melanocytes, was tested in a phase II study recently (NCT02363283). Binding to the NMB leads to internalization of the conjugate and release of the drug in the cells.

Interfering with epigenetic dysregulation represents the most recent approach in UM treatment; trials are ongoing with the HDAC inhibitors vorinostat (NCT02068586, NCT01587352) and entinostat (PEMDAC trial with pembrolizumab, entinostat, NCT02697630), and the BRD4 inhibitor PLX51107 (NCT02683395).

## 3. Uveal Melanoma: Urgent Need for Progress

Basic requirements for optimal management of rare cancers include early diagnosis, referral to a specialized centre, establishment of the therapeutic strategy by a specialist multidisciplinary team (a multidisciplinary tumour board), and access to appropriate treatments at all stages. The major issues in UM are late diagnosis or misdiagnosis, non-expert management, high risk for distant recurrence, and absence of effective treatment in the metastatic setting.

Because of limited published studies, insufficient knowledge, and unshared experience, there is a considerable risk for UM patients to be managed outside expert centres. With UM being a rare melanoma, numbers of tumours are unnecessarily checked for BRAF mutational status, and many patients undergo 18F-Fluorodeoxyglucose PETCT and receive immunotherapy as for cutaneous melanoma.

To avoid these pitfalls, and compared to frequent cancers, we need to:

-expand preclinical research in UM: basic research deciphering UM biology, establishing animal models [45,46], recapitulating patient tumours’ characteristics; and translational research discovering biomarkers and new drugs. This process is even more crucial as setting up clinical trials in an orphan disease remains an international challenge.

-arouse scientific interest and attract pharmaceutical companies to allocate resources to UM-dedicated research and clinical trials. Moreover, we should allow specific cohorts of rare cancers with no reference treatment and a dismal prognosis to participate in early phase and molecular-driven clinical trials (i.e., slots for UM patients).

-develop training (for students, for nurses) and information (for patients, for general practitioners) with adequate and specific communication tools.

-act jointly with health authorities in each European Union member state to foster early approval and availability of drugs in such orphan cancers.

## 4. What is the EURACAN Organisation?

European Reference Networks (ERNs) are virtual networks launched by the European Commission in 2017 with the support of the EU Health Programme. They involve over 300 healthcare providers within 24 ERNs (four dedicated to rare cancers and predisposing conditions) in 26 member states to share expertise and improve access to care for patients across the European Union, especially for complex or rare medical diseases that require highly specialized healthcare and a concentration of knowledge and resources. ERNs work on a range of thematic issues including bone disorders, childhood cancer, and immunodeficiency.

The EUropean Rare Adult solid CAncer Network (EURACAN) is the European Reference Network dedicated to rare adult solid cancers. It aims to improve the quality of care and outcome of patients with rare adult solid cancers in the EU by optimizing diagnostic and therapeutic management, knowledge, research, and communication on all adult solid rare cancers for patients, families, physicians, and all stakeholders.

The network gathers 66 health care providers in 17 European countries, identified based on documented expertise, experience treating rare cancers, and endorsement by their member state. It groups all rare solid adult cancers into ten “domains” corresponding to the RARECARE list of rare cancers based on the International Classification of Diseases for Oncology (ICD-O): sarcomas; rare gynaecologic tumours; rare neoplasms of the male genital organs and of the urinary tract; neuroendocrine tumours; rare neoplasms of the digestive tract; rare neoplasms of endocrine organs; rare neoplasms of the head and neck; rare neoplasms of the thorax; rare skin cancers and eye melanoma; rare neoplasms of the brain and spinal cord.

EURACAN invited 16 associated partners from major scientific societies and key stakeholders: European Association of Neuro-Oncology (EANO), European CanCer Organisation (ECCO), European Neuroendocrine Tumours Society (ENETS), European Organisation for Research and Treatment of Cancer (EORTC), European Society of Gynaecological Oncology (ESGO), European Society of Medical Oncology (ESMO), European School of Oncology (ESO), European Society of Surgical Oncology (ESSO), European Society for Radiotherapy and Oncology (ESTRO), French Sarcoma Group-Bone Tumour Study Group (GSF-GETO), Joint Action on Rare Cancer (JARC), Organisation of European Cancer Institutes (OECI), French National Network for the Treatment of Rare Peritoneal Surface Malignancies (RENAPE), Network for the management of Thymic tumours and cancer (RYTHMIC), University of Milan (UNIMI), University Claude Bernard Lyon (UCBL) and 13 leading patient advocacy organisations in each domain: Italian Association for Laringectomy (AILAR), Association for Multiple Endocrine Neoplasia Disorders (AMEND), Digestive Cancer Europe (DiCE), European cancer Patient Coalition (ECPC), EUROpean Rare DISease (EURORDIS), International Brain Tumour Alliance (IBTA), International Neuroendocrine Cancer Alliance (INCA), Neuroendocrine tumour (Net) patient foundation, Northern Ireland Rare Disease Partnership (NIRDP), Melanoma Patient Network Europe (MPNE), Salivary Gland Cancer UK (SGC) , Sarcoma PAtient Euro Net (SPAEN), Thyroid Cancer Alliance (TCA) ) to participate in the different network bodies.

## 5. EURACAN Missions in Uveal Melanoma Domain

Groups of experienced specialists gathered in national and international networks can provide collaborative efforts to better understand the biology of rare diseases and to develop innovative treatment approaches. EURACAN provides an infrastructure integrating national networks and European health care providers (HCPs) with a high level of expertise but also European patient advocacy groups (ePAGS) that together act as focal points for research on rare tumours and keys in the dissemination of knowledge and information.

Because of the rarity of UM, medical expertise may be difficult to access, and patients may search for cross-border second opinions and appropriate care from country to country. Inappropriate management at early stages may result in an increased risk of metastasis and fatal outcome. Screening by ophthalmologists and referral to expert ophthalmologists is crucial to avoid any late diagnoses.

**Figure 1:**
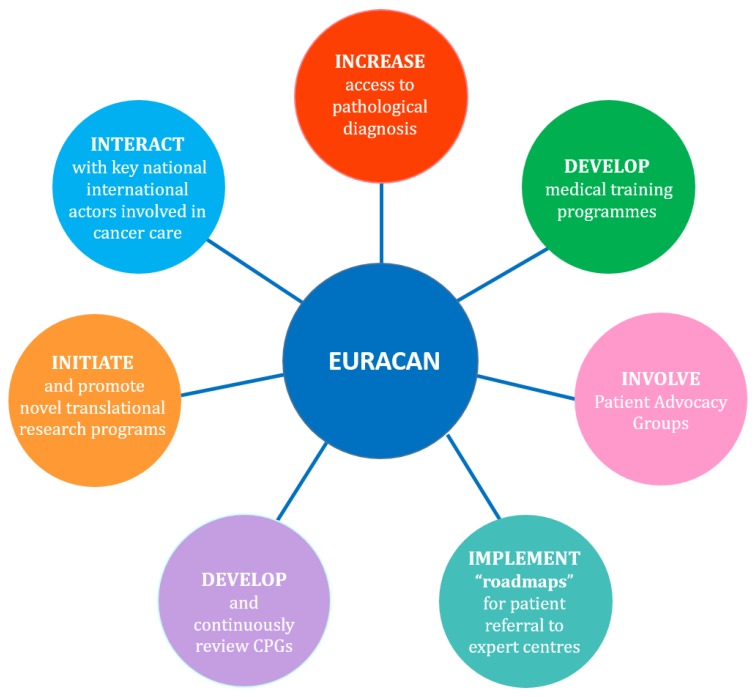
Objectives of the European Reference Network for Rare Adult solid Cancers (ERN-EURACAN).

Within the European Reference Network EURACAN, the G9 domain is dedicated to rare skin cancers and uveal melanomas. The objectives of the network for the UM subdomain include the description of the organisation of UM care in each participating country, the development and review of European clinical practice guidelines (CPGs), and the implementation of “roadmaps” for referral and self-referral of patients to expert centres (Figure 1).

In the context of the lack of funding for research in rare cancers, EURACAN will also participate in the development of innovative strategies, providing the frame for the initiation and the promotion of novel translational and clinical research programs through international collaborations and associated tools (e.g., multinational databases, registries and tumour banks).

To increase and harmonise the quality of care, a web-based-platform provided by the European Commission and customized to fit all domain needs will be used for multidisciplinary discussions. In parallel, EURACAN will develop medical training programs accessible to physicians and ePAGs representatives and will interact with patient advocacy groups to increase their involvement and assist them in the wide dissemination of communication and educational tools. In 2015, the patient organisation Melanoma Patient Network Europe (MPNE) created a new entity dedicated to rare melanomas and had already begun to organize meetings focusing on UM (www.melanomapatientnetworkeu.org).

Moreover, EURACAN will initiate and promote collaboration with pharmaceutical companies by networking for industry sponsorship for rare cancers, helping to conceptualize clinical trials dedicated to UM with adapted design and consensual endpoints, and facilitating the identification of participating sites in each country and the enrolment of patients in a short period of time. The interaction with health authorities and regulatory agencies could help to successfully drive these efforts to accelerate registration of any active agent as an orphan drug at the European level. Finally, in collaboration with academic societies and international groups, EURACAN provides the integration of clinical care and research programs dedicated to UM patients in order to increase the quality of care for rare cancers in all European countries.

## 6. Conclusions

The biology of UM differs from that of CM, and this rare melanoma deserves specific means and dedicated funding to develop international collaborations and global integration of research and clinical care, with the aim of ensuring equal access to high quality care and innovation to all UM patients. The ERN EURACAN represents a unique European effort to address this urgent and unmet clinical need.
